# Masquelet Technique for Treatment of Posttraumatic Bone Defects

**DOI:** 10.1155/2014/710302

**Published:** 2014-02-06

**Authors:** Tak Man Wong, Tak Wing Lau, Xin Li, Christian Fang, Kelvin Yeung, Frankie Leung

**Affiliations:** ^1^Department of Orthopaedics and Traumatology, The University of Hong Kong, Queen Mary Hospital, 102, Pokfulam Road, Hong Kong; ^2^Shenzhen Key Laboratory for Innovative Technology in Orthopaedic Trauma, The University of Hong Kong-Shenzhen Hospital, 1 Haiyuan 1st Road, Futian District, Shenzhen, China

## Abstract

Masquelet technique, which is the use of a temporary cement spacer followed by staged bone grafting, is a recent treatment strategy to manage a posttraumatic bone defect. This paper describes a series of 9 patients treated with this technique of staged bone grafting following placement of an antibiotic spacer to successfully manage osseous long bone defects. The injured limbs were stabilized and aligned at the time of initial spacer placement. In our series, osseous consolidation was successfully achieved in all cases. This technique gives promising result in the management of posttraumatic bone defects.

## 1. Introduction

Segmental bone defects resulting from traumatic injuries are complicated problems with significant long-term morbidity. Historically, due to the difficulty in managing segmental long bone defects, amputation was the preferred treatment. Limb salvage has been developed over the last half century. During World War II, massive cancellous bone autograft has been the mainstay of treatment [1, 2]. The use of the Ilizarov technique, vascularized fibular grafts, and acute limb shortening have been used previously to address defects of various lengths. Traditional bone graft techniques are limited by uncontrollable graft resorption, even when the recipient site is well vascularized [3]. More recently, the use of an antibiotic cement spacer followed by grafting within this space confirmed by an induced biomembrane has been described as a potential treatment strategy [4, 5]. This paper describes a series of patients at our institution successfully treated with this technique.

## 2. Patients and Methods

Between 2009 and 2012, all patients admitted with post-traumatic bone defects and managed by Masquelet technique (Table 1) were recruited. The patients were evaluated for injury type, location, soft tissue condition, length of bone defect, antibiotic used, and duration of cementation. Moreover, the type of fixation, presence of infection, and current state of all patients were recorded.

## 3. Surgical Technique

**Figure 1:**
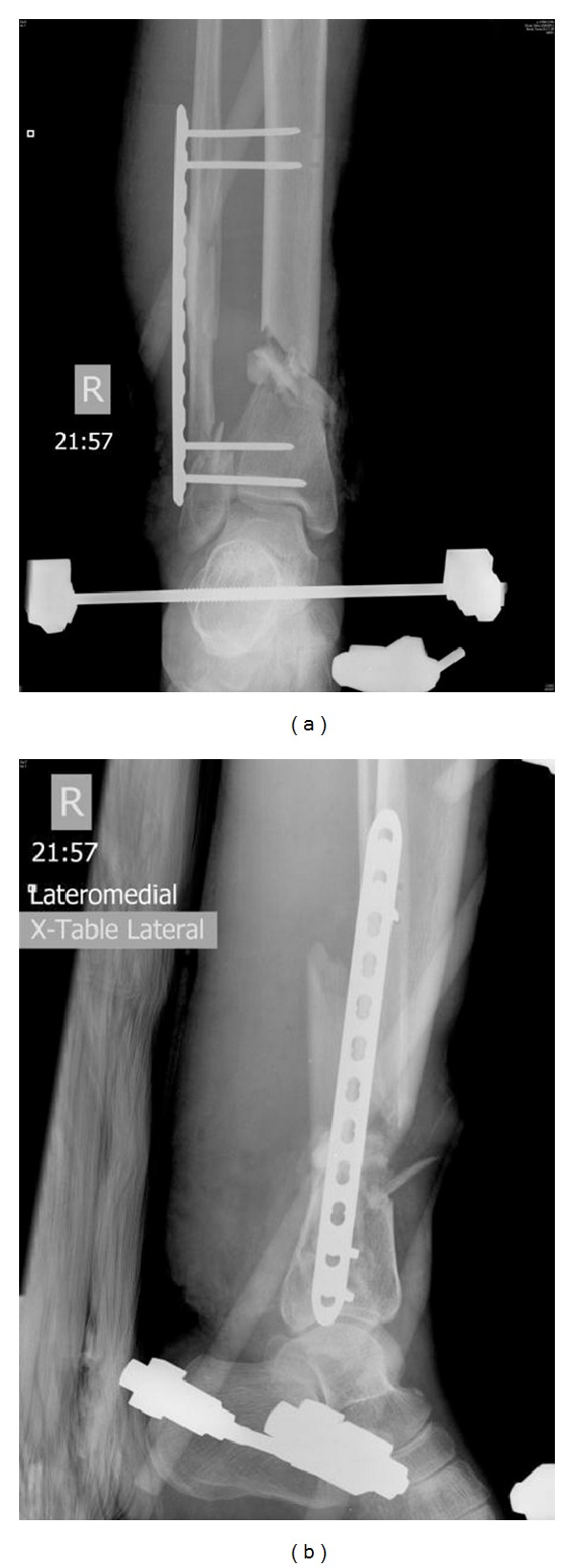
AP (a) and lateral (b) radiographs of an open fracture right distal tibia Gustilo Type IIIA at admission. It was initially debrided, stabilized, and shortened with an external fixator, leaving a defect over right distal tibia.

**Figure 2:**
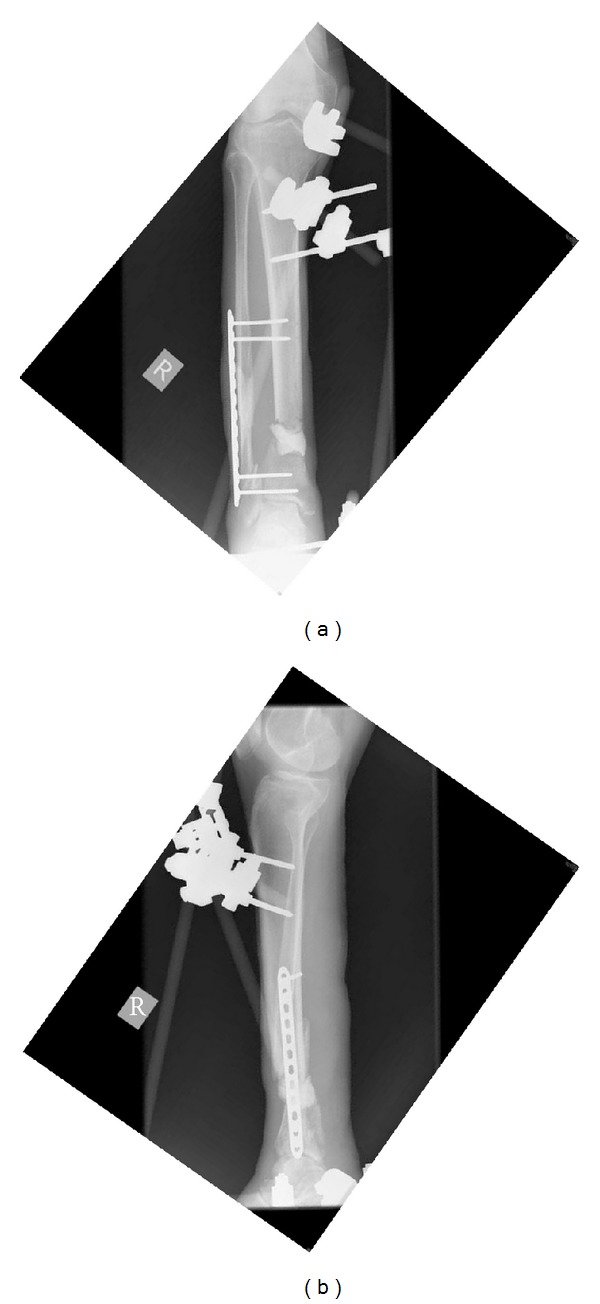
AP (a) and lateral (b) radiographs showing fixation with external fixator and screws and placement of antibiotic cement spacer into the defect after the wound had been adequately debrided.

**Figure 3:**
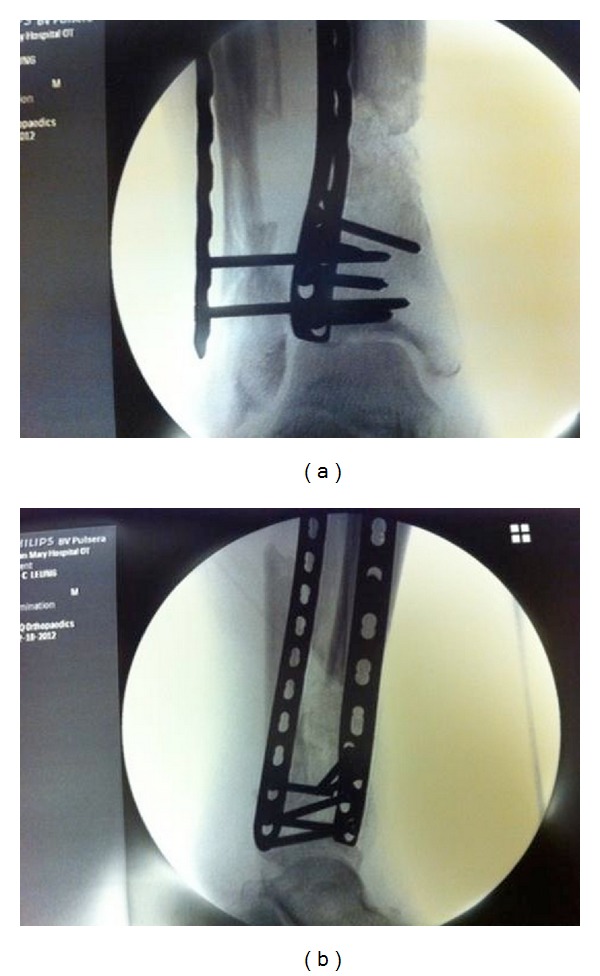
AP (a) and lateral (b) fluoroscopic images showed the cement spacer being removed and the defect filled with cancellous autograft harvested from iliac crest.

During the first stage, the operative extremity was prepared and draped in the usual sterile fashion. The area of bone loss was carefully debrided and irrigated. Debris and nonviable tissues were removed. Careful dissection was then performed down to the fracture site and the fracture ends were identified and debrided again. Based on preoperative templating, the length, alignment, and rotation of the injured limb were obtained. Method of fixation depended on the fracture type and location. For open fracture, with significant defect, external fixator was used temporarily (Figure 1). Once acceptable reduction was achieved (ensuring anatomic length, alignment, and rotation), fixation was undertaken. Once fixation had been achieved, attention was then turned to the bone defect. The defect was measured and filled with a polymethylmethacrylate (PMMA) bone cement spacer. We preferred to use 2 g vancomycin or gentamicin per 40 g of cement prepared (Figure 2). The second stage of bone grafting was performed 4–12 weeks after the first surgery. The bone graft was harvested from the iliac crest. The fracture was approached through the previous incision and careful dissection was performed down to the defect. The biomembrane encapsulating the cement spacer was carefully incised. Once exposed, the cement spacer was removed. Once the cement spacer was removed, the biomembrane capsule was irrigated to remove any residual debris. With the defect being open, bone graft was placed to fill the entire defect (Figure 3). The defect should be completely filled but not overstuffed. Once the defect was filled, the biomembrane was closed with absorbable suture.

## 4. Results

A total of 9 consecutive patients were identified within the time period. The series included 7 men and 2 women, with a mean age of 53 (27–79). The bone defects were located at tibia (3 cases,) the femur (2 cases), the humerus (1 case), the olecranon (2 cases), and calcaneum (1 case). Four cases were closed fracture but complicated with infection or nonunion. The other five cases were open fractures with bone loss (Gustilo Classification Type II or IIIA).

**Figure 4:**
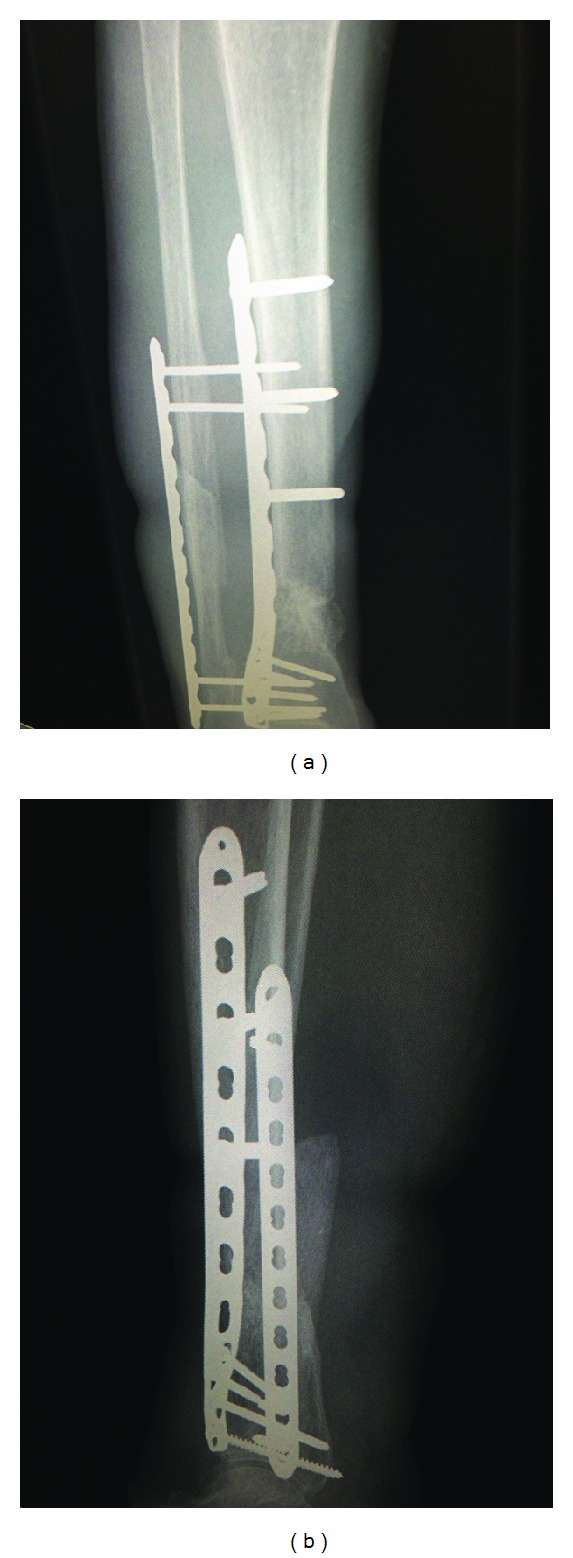
AP (a) and lateral (b) radiographs taken 6 months later showing osseous consolidation.

The length of bone defect ranged from 2 cm to 8 cm. The antibiotics used for cement spacer were either gentamicin or vancomycin. The mean interval between the first-stage and second-stage surgeries was 48.5 days (30–57). All affected limbs were fixed with screw and plate construct. All patients demonstrated radiographic consolidation over the defect after treatment (Figure 4). No complication was reported in the series.

## 5. Discussion

Treatment of large segmental bone defects can be challenging for orthopaedic surgeons. Masquelet et al. [6] described a procedure combining induced membranes and cancellous autografts. Bone grafting of these defects is often delayed after primary fixation to allow soft tissue healing, decrease the risk of infection, and prevent graft resorption [7]. In traumatic wounds, antibiotic impregnated cement beads or spacers are often used for local antibiotic administration to the soft tissue bed. In addition, the advantages of inserting such a spacer include maintaining a well-defined void to allow for later placement of graft, providing structural support, offloading the implant, and inducing the formation of a biomembrane. Masquelet and Begue proposed that this membrane prevents graft resorption and improves vascularity and corticalization. It has been described that, after the initial placement of the antibiotic impregnated spacer, an interval of 4 to 5 weeks is needed for development and maturation of a biologically active membrane that is suitable for grafting. The spacer also maintains the defect and inhibits fibrous ingrowth [5].

Recent literature has shown that this biomembrane can be 0.5 to 1 mm thick [8] and has been described as both hyper-vascular and impermeable [9]. Viateau et al. [10] studied this technique in a sheep model and found that the membrane alone was inadequate to heal a large defect. But when autologous bone graft was placed within the membrane, all the defects went on to heal. The technique of inducing a biomembrane at the site of an osseous defect with staged grafting has been described in case reports for defects of various sizes and in various locations throughout the skeletal system. The mechanism of action of induced membranes in bone repair was studied recently by Aho and his colleagues [11]. They found that the one-month-old membrane has higher osteogenesis-improving capabilities compared to two-month-old membrane; they concluded that optimal time for performing second-stage surgery may be within a month after implantation of foreign material [11]. In our series, the mean interval between the first and second surgeries is 43.5 days, which is comparable to other studies.

Pelissier et al. [9] reported that the induced membranes secrete growth factors including vascular and osteoinductive factors and could stimulate bone regeneration. Biau et al. described the management of a 16 cm defect in the femur of a 12-year-old child who had been diagnosed with Ewing's sarcoma and required resection of a large segment of his femur. The segmental defect was stabilized with an intramedullary nail and then maintained with an antibiotic spacer until later grafting and eventual healing [12]. However, Accadbled et al. reported their 3-case study showing that reconstruction of the femur seems to be specifically associated with a risk of graft resorption. Accadbled et al. [13] reported a case using a cage and nail construct, resulting in successful eradication of methicillin-resistant staphylococcus aureus infection and reconstitution of a 17 cm diaphyseal defect in the tibia [14]. As mentioned, the technique has been used to address bone loss in areas other than long bones. Huffman et al. [15] reported use of the technique in a significant area of bone loss in the midfoot of a patient who had sustained multiple gunshot injuries. The original description of this technique described stabilization of the bone with an external fixator, but as noted, other means of fracture fixation have been used with success. Apard et al. [16] reported a series of 12 patients who presented with 6 cm segmental defects in the tibia, all of whom were initially fixed with an intramedullary nail. They reported healing following the second-stage procedure in 11 of 12 patients at an average of 4 months [16]. To our knowledge, no study has evaluated the optimal bio-mechanical environment for such a technique; rather each fracture is “bridged” according to the treating surgeon's assessment of the fracture. A potential effect of a construct that is too rigid may be stress shielding near the plate, reducing integration of the bone graft near the implant. This does not preclude bony union but may increase time to osseous consolidation and affect the radiographic appearance of the defect. The technique as described by Masquelet and Begue [5] relied on the placement of morselized cancellous autograft harvested from the iliac crests within the biomembrane lined defect. If this amount is not sufficient, demineralized allograft is added to the autograft in a ratio that does not exceed 1 : 3 [5]. In our study, we used autograft harvested mainly from iliac crest, without any allograft. Biau et al. [12] used both iliac crest corticocancellous autograft and a medial tibial cortical strut autograft to fill their large defect. Use of cancellous autograft from the femoral canal has also been described, and evidence exists to show that levels of many growth factors (fibroblast growth factor-*α*, platelet derived growth factor, insulin-like growth factor 1, TGF-1, and BMP-2) in femoral cancellous bone are present in higher concentrations than they are in iliac crest and platelet preparations [17]. In our series, we used Masquelet technique to treat post-traumatic bone defect successfully. Further research and clinical series will hopefully elucidate the grafting components necessary to optimise healing in these patients.

## 6. Conclusion

The technique of delayed bone grafting after initial placement of a cement spacer provides a reasonable alternative for the challenging problem of significant bone loss in extremity reconstruction. This technique can be used in either an acute or delayed fashion with equally promising results. The bioactivity of the membrane created by filling large bony defects with cement leads to a favourable environment for bone formation and osseous consolidation of a large void. As this technique becomes more widely applied, the answer to which graft substances to place in the void may become clearer. Increasing clinical evidence will also help support the use of this technique in treating segmental bone loss.

## Figures and Tables

**Table 1:** Patient demographics.

Patient number/sex/age (y)	Type of injury	Fracture type	Soft tissue condition	Indication	Bone defect, length (cm)	Spacer	Definite fixation	Current State	Duration of cementation (days)
1/M/60	Fracture distal humerus	Closed	Wound contaminated	Postoperative wound infection	2 cm	Gentamycin	Plate and screw	Bone grafted and healed	50

2/M/60	Fracture olecranon	Open Gustilo II	Viable, not contaminated	Bone loss	2 cm	Vancomycin	Plate and screw	Bone grafted and healed	57

3/M/28	Fracture distal femur	Open Gustilo IIIA	Gross infection, contaminated	Postoperative wound infection	8 cm	Gentamycin	Plate and screw	Bone grafted and healed	48

4/F/79	Fracture left olecranon	Closed	Infection deep to joint, contaminated	Postoperative wound infection	4 cm	Gentamycin + vancomycin	Plate and screw	Bone grafted and healed	53

5/M/53	Fracture tibial plateau	Closed	Deep infection extending to knee joint, contaminated	Postoperative wound infection	4 cm	Gentamycin + vancomycin	Plate and screw	Bone grafted and healed	48

6/M/48	Fracture os calcis	Open Gustilo II	Soft tissue viable, not contaminated	Bone loss	2 cm	Gentamycin	Plate and screw	Bone grafted and healed	30

7/M/61	Fracture distal femur	Closed	Viable, not contaminated	Nonunion	3 cm	Gentamycin + vancomycin	Plate and screw	Bone grafted and healed	43

8/F/27	Fracture distal tibia	Open Gustilo II	Not contaminated	Bone loss	2 cm	Gentamycin	Plate and screw	Bone grafted and healed	59

9/M/60	Fracture distal tibia	Open Gustilo IIIC	Minimal contaminated	Bone loss	4 cm	Vancomycin	Plate and screw	Bone grafted and healed	49
